# Research on the Asymmetric Phenomenon of Voltage Polarity Based on Dielectric Wetting

**DOI:** 10.3390/ma17112717

**Published:** 2024-06-03

**Authors:** Yuxing Ye, Hui Jin, Meng Zhao, Fengying Xu, Zhuo Jiang

**Affiliations:** 1 College of Engineering, South China Agricultural University, Guangzhou 510640, China; yuxing_ye1215@163.com (Y.Y.); xu_fy@scau.edu.cn (F.X.); 2 Micro-Nano Tech Center, Bioland Laboratory, Guangzhou 510000, China; huihuijin2@126.com; 3 College of Food Science, South China Agricultural University, Guangzhou 510640, China

**Keywords:** dielectric wetting, voltage polarity, asymmetric electrowetting, leakage current, droplet contact angle

## Abstract

The present research investigated the voltage polarity asymmetry phenomenon based on dielectric wetting. In an ITO–hydrophobic layer–droplet setup, three reagents with different pH values (3.96, 7.0, and 10.18), two types of hydrophobic materials (AF1601 and 6%T6), and two different thicknesses (340 nm and 2.5 μm) of each material were systematically investigated. The results show that the thickness of the hydrophobic dielectric layer and the pH of the droplets had a significant impact on the droplet contact angle variation with the voltage. The contact angle on the thick hydrophobic dielectric layer followed the Lippmann–Young equation as the voltage changed. The angle of the thin hydrophobic dielectric layer was affected by its own properties and the type of droplet, which led to the occurrence of voltage polarity asymmetry of the electrowetting phenomenon. After further investigation of this phenomenon, it was found that it mainly accounted for the decrease in electric field strength at both ends of the droplet, which was caused by electrochemical reactions and changes in circuit resistance. The leakage current is an important indicator, and this phenomenon can be prevented by increasing the thickness of the hydrophobic dielectric layer.

## 1. Introduction

**Figure 1:**
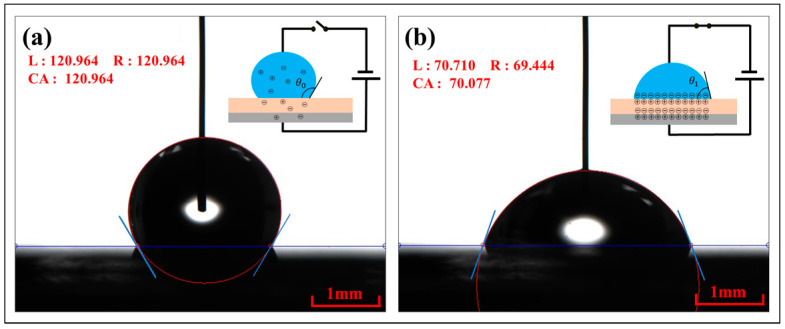
Schematic diagram of EWOD. (**a**) The state of the droplet before charging. (**b**) The state of the droplet after charging.

The past decade has witnessed the breathtaking advancement of the electrowetting-on-dielectric (EWOD) technique [1]. EWOD refers to the phenomenon where the contact angle of droplets [2] changes under the action of an electric field, as shown in Figure 1. Before applying voltage, there are only trace amounts of free electrons in the droplet and the substrate. The contact angle of liquid droplets is relatively large. Under the action of the electric field, the same amounts of dissimilar charge accumulate at the interface between the droplet and the dielectric layer, as well as between the dielectric layer and the electrode layer, forming a double electric layer [3]. With an increase in the electric field strength, the surface tension of the solid–liquid interface of the droplet decreases [4], and the contact angle decreases while the wettability increases. In this process, *θ*_0_ represents the initial contact angle, *θ_1_* represents the energized contact angle, and *θ*_0_ > *θ*_1_. L and R represent the contact angles of the left and right sides of the droplet, respectively, and CA represents the average value of the contact angles of the left and right sides of the droplet.

Based on the EWOD principle, a lot of applications have been developed, including chip laboratory equipment [5,6,7,8,9], optical lenses [10,11], displays [12,13], and energy-harvesting systems [14,15,16]. In order to actuate droplets more efficiently, rapidly, and accurately, it is important and necessary to explore the relationships between the contact angle and the applied voltage. Among them, variation in the contact angle with the voltage follows the Lippmann–Young equation [17]:

(1) $$cos\theta =cos\theta_{0}+\frac{\varepsilon_{0}\varepsilon_{d}}{2d\gamma_{LG}}V^{2},$$

In Equation (1), *θ*_0_ is the initial contact angle, *ε_0_* is the vacuum permittivity [18], *ε_d_* is the permittivity of the dielectric layer, *d* is the thickness of the dielectric layer, *V* is the voltage, and *γ_LG_* is the surface tension of the gas–liquid interface field. Theoretically, the droplet contact angle decreases with the voltage, and the angle is not affected by the polarity of the voltage. However, in practical applications, researchers have found that the contact angle of droplets does not decrease infinitely with the voltage, and there is a phenomenon of contact angle saturation [19]. The droplet contact angle is also affected by voltage polarity, and there is an asymmetric electrowetting phenomenon [20,21,22] with respect to the axis *V* = 0.

At present, the various explanations for contact angle saturation remain controversial, including the ionization of air near the contact line [23], the resistance of droplets [24], the charge capture of the dielectric layer to generate charge shielding [25], and the surface tension limit of the solid–liquid interface [26]. Meanwhile, a lot of research on the asymmetric electrowetting phenomenon has attracted the attention of many scholars. Seyrat and Hayes [27] observed an asymmetric electrowetting phenomenon in 0.5 μm thin fluorine-containing polymers, and they speculated that the phenomenon may be related to the nano-porosity of fluorine-containing polymers and the existence of O atoms in their structure. However, they did not conduct further theoretical analysis. Hyejin Moon et al. [28] found a similar phenomenon in 1 μm thick fluorine-containing polymers, which was attributed to the preferential adsorption of hydroxide ions at the Teflon–water interface. However, it cannot explain why acidic droplets also exhibit this phenomenon. Mehdi Khodayari et al. [29] noticed that after the electrochemical reaction occurred on an electrowetting substrate, the potential of the auxiliary electrode decreased, resulting in asymmetric electrowetting. However, not all electrodes undergo electrochemical reactions. Banpurkar et al. [30] and Wu et al. [31] believe that this phenomenon is related to charge trapping on the surface of Teflon, and the amount of charge injected on the surface of Teflon is also controlled by the pH of water. However, the properties of hydrophobic materials and droplets have not been further explored.

In summary, the phenomenon of asymmetric electrowetting is still not fully understood, as existing theories do not have universality and can only explain some phenomena in specific cases. The thickness and type of the hydrophobic dielectric layer, the ion concentration of the droplet, and the acidity and alkalinity of the droplet all have varying degrees of influence on this phenomenon. In addition, in practical applications based on the EWOD principle [32], droplet electrolysis and dielectric layer breakdown often occur. The reason for this phenomenon is likely to be related to the asymmetric voltage polarity electrowetting phenomenon. Therefore, studying the phenomenon of voltage polarity asymmetric electrowetting is of great significance for understanding and applying the EWOD principle.

The main content of the present study consists of three parts: (1) The electrowetting behavior of hydrophobic dielectric layers at the nanoscale and with a micrometer thickness is explored, and the causes of asymmetric electrowetting phenomena are identified. (2) The effects of different pH solutions (citric acid and sodium citrate buffer, ultrapure water, sodium carbonate, and sodium bicarbonate buffer) on the dielectric wetting behavior are explored. (3) The influence of different hydrophobic materials (6%T6 and AF1601) on electrowetting behavior is also explored.

## 2. Materials and Methods

### 2.1. Materials and Reagents

The electrode layer adopted a typical commercial ITO (indium tin oxide) glass plate with a size of 95 × 56 mm and a thickness of 1.1 mm. The thickness of the ITO film was 185 nm, and the magnitude of the square resistance was about 7 Ω/Sq. Two types of hydrophobic materials were applied: AF1601 and 6%T6. Among them, AF1601 fluorinated resin solution was obtained from Teflon Company (Wilmington, DE, USA), and 6%T6 fluorinated resin solution was obtained from Fu ze New Materials Company (Shanghai, China). Citric acid, sodium citrate, sodium carbonate, and sodium bicarbonate were sourced from Aladdin Biochemical Company (Shanghai, China).

#### 2.1.1. Preparation of Hydrophobic Dielectric Layer

We used the following process steps to prepare the hydrophobic dielectric layer on these ITO glasses surface: (1) the ITO glass was placed in an ultrasonic cleaning machine (KS-500DE, ShuMei, Kunshan, China) to clean (industrial cleaning agent for 12 min; ultrapure (UP) water for 12 min and ethanol for 12 min, respectively). (2) After cleaning, the ITO glass was put into a constant-temperature drying box (DHG-9023A, Shen Xian, Shanghai, China) to dry (60 °C for 10 min). (3) The cleaned and dried ITO glass was placed into the spin coater (EZ4, LEBO, Jiangsu, China) and spin-coated with 500 microliters of AF1601 or 6%T6 fluorinated resin solutions, respectively, to obtain hydrophobic layers with different thickness (the spin-coating speed for 340 nm thickness AF1601 was set as 500 rmp/20 s, followed by 2000 rmp/60 s, and for the 2.5 μm thickness AF1601, one was set as 500 rmp/10 s only; while the coating speed for 340 nm 6%T6 thickness was set to 500 rmp/20 s, followed by 1199 rmp/60 s, and for the 2.5 μm thickness 6%T6, one was set to 500 rmp/10 s). (4) After the completion of the spin coating of the ITO glass, it was placed into a constant-temperature drying oven for drying. The drying temperature was set to 120 °C and lasted for 40 min. The film thickness of the hydrophobic dielectric layer was measured using a Stepping instrument (Dektak-XT, Bruker, Luken, Germany), and the surface structure of the hydrophobic materials was examined using scanning electron microscopy (Verios-460, FEI, Hillsborough, OR, USA). During the measurement process, the ambient temperature was maintained at 24 ± 5 °C.

#### 2.1.2. Experimental Reagent Configuration

Three different pH reagents were required in the experiment: 0.1 mol/L citric acid and sodium citrate buffer (CA-SCB) [33], UP water, and 0.1 mol/L sodium carbonate and sodium bicarbonate buffer (SC-SBB) [34]. To obtain them, CA-SCB and SC-SBB were prepared using commercial formulas, and the specific parameters are shown in Table 1 [35,36]. UP water was obtained using an UP water system (MolliQ-IQ700, Merck, Kenilworth, NJ, USA), and the actual pH and conductivity of the reagent were measured using a pH meter (PHS-3E, Lei Ci, Shanghai, China) and a conductivity meter (DDS-11A, Yue Ping, Shanghai, China).

### 2.2. Experimental Methods

#### 2.2.1. Contact Angle Measurement

**Figure 2:**
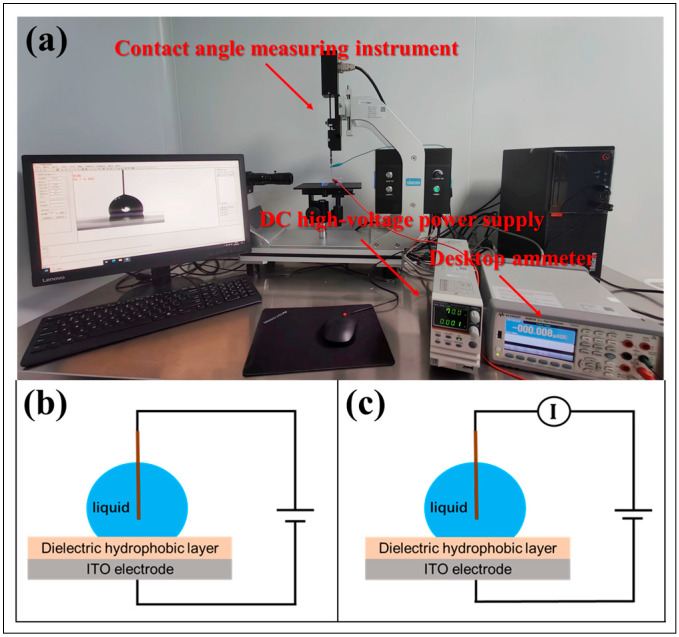
Contact angle and leakage current testing system and model. (**a**) Physical diagram of the testing system. (**b**) Electric contact angle test model. (**c**) Leakage current test model.

Based on the principle of the sessile drop method [37], a contact angle measuring instrument (SINDIN SDC-200S, Sheng Ding, Foshan, China) and its accessories were used to complete the measurement of the droplet contact angle, and a DC high-voltage power (PSW-800, GWINSTEK, Taiwan, China) supply was used to actuate the droplet. The test system and model are shown in Figure 2a,b. The two ends of the power supply were connected to an ITO electrode and a tungsten wire (length 30 mm, diameter 0.1 mm), respectively. The tungsten wire was inserted vertically into the droplet at about a 1/3 depth from the bottom of the droplet. When a positive voltage was applied, the positive pole of the power supply was connected to the ITO electrode, and the negative pole was connected to a tungsten wire. When negative voltage was applied, the power cord was reversed. It should be noted that the power supply used in this study was a DC power supply that did not involve AC.

In the experiment, a pipette was used to add the samples, and the volume of each droplet was maintained at 5 μL. The tungsten wire was replaced at every 10 points to reduce the influence of the auxiliary electrode on the experiment [29]. When loading the droplets, the position of each drop was not repeated to prevent the charge shielding of the hydrophobic dielectric layer. Three groups of parallel experiments were conducted for each voltage gradient, the ITO glass was replaced when the voltage polarity was changed, and the ambient temperature was maintained at 24 ± 2 °C.

#### 2.2.2. Leakage Current Measurement

A desktop digital multimeter (34410A, Keysight, Santa Rosa, CA, USA) was selected to complete the measurement of the leakage current of the hydrophobic dielectric layer [38,39,40,41,42]. Before the experiment, it was connected in series with the DC high-voltage power supply in the circuit, as shown in Figure 2c. The contact angle deviation or saturation matters a lot, which occurs in both DC and AC drives, and this phenomenon is much more obvious in the DC drives mode. For the convenience of research, we focus our study on the effect of DC drives mode in this article. During the experiment, the droplets were energized at a gradient of 10 V (thin hydrophobic dielectric layer)/20 V (thick hydrophobic dielectric layer) and maintained for 30 s, and the current value was recorded. When the set voltage value (70 V/150 V) or the current limit value of the ammeter (1.8 mA) was reached, the voltage was stopped.

## 3. Results

### 3.1. Structure of the Hydrophobic Layer

Based on the EWOD principle, the phenomenon of electrowetting models with different hydrophobic materials and droplet compositions varies. In this study, four different EWOD models were tested, as shown in Table 2, including two different thicknesses, two types of hydrophobic dielectric layers, and three different reagents. Among them, ITKAD represents the electrowetting model composed of ITO–thick–AF1601–droplet. ITNAD represents the electrowetting model composed of ITO–thin–AF1601–droplet. ITK6D represents the electrowetting model composed of ITO–thick–6%T6–droplet. ITN6D represents the electrowetting model composed of ITO–thin–6%T6–droplet. In addition, thickness (δ), resistivity (*ρ*), pH, and conductivity (σ) were measured using corresponding equipment, and each parameter was measured at no less than four data points.

**Figure 3:**
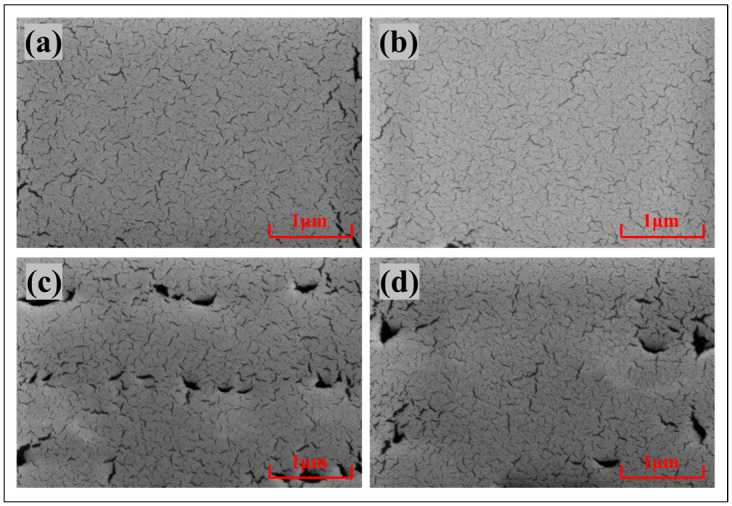
SEM of hydrophobic materials. (**a**) AF1601–thin; (**b**) 6%T6–thin; (**c**) AF1601–thick; (**d**) 6%T6–thick.

In order to further investigate the properties of the material, the surface morphology of the hydrophobic layer was observed using a scanning electron microscope, and the results are shown in Figure 3. It can be observed that the surfaces of the 6%T6 and AF1601 hydrophobic materials are not completely dense with cracks and voids present in both. By comparing these two hydrophobic materials, it can be observed that AF1601 has more cracks than 6%T6. Although the thick hydrophobic layer appears to have larger pores, the pore size is smaller than the thickness, and the surface is still dense. The pore size and thickness of the thin hydrophobic layer are equivalent, and it may not be dense, as shown in Figure 3c,d.

### 3.2. Contact Angle

#### 3.2.1. Thick Hydrophobic Dielectric Layer

**Figure 4:**
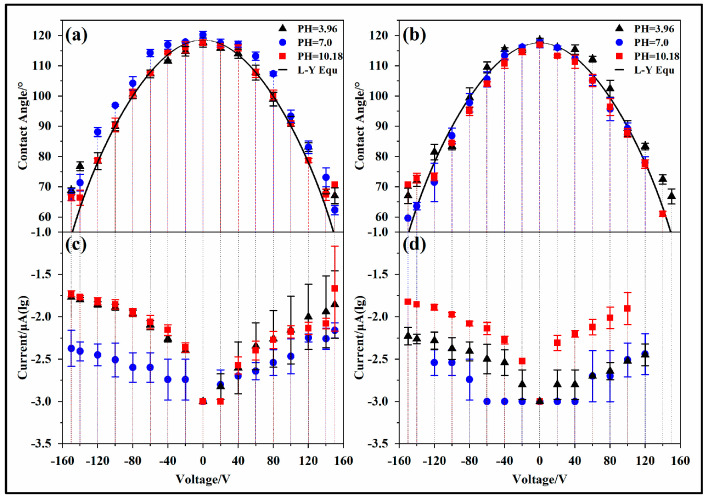
Effect of voltage on droplet contact angle and leakage current. Electrowetting models: (**a**,**c**) ITKAD and (**b**,**d**) ITK6D.

Regarding the experimental electrowetting models ITKAD and ITK6D, the droplet contact angle varied with the voltage (−150~150 V) following the Lippmann–Young equation, as shown in Figure 4a,b, and the measured contact angle was basically consistent with the theoretical value. In the figure, the black solid line represents the fitted value of the Lippmann–Young equation; the black solid triangle indicates that the tested droplets are CA-SCB with a pH of 3.96; the blue solid circle indicates that the tested droplet is UP water with a pH of 7; and the red solid square indicates that the tested droplets are SC-SBB with a pH of 10.18. It is worth noting that when the voltage was high, the droplets bounced, and, thus, the contact angle could not be measured. Therefore, some data points are missing in Figure 4.

According to the data, when the applied voltage was less than 140 V, the measured contact angle did not differ from the theoretical value by more than 5°. When the applied voltage was greater than 140 V, the measured value of the droplet contact angle differed from the theoretical value by about 10°. At the same time, by comparing acidic (pH = 3.96) and alkaline (pH = 10.18) solutions and by comparing solutions with high ion concentrations (0.1 mol/L) and UP water, it was found that there was no significant difference in the droplet contact angle with changes in the voltage, as shown in Figure 4a,b. Although the leakage current of the hydrophobic dielectric layer slowly increased with voltage, it was at the nanoampere level, as shown in Figure 4c,d.

#### 3.2.2. Thin Hydrophobic Dielectric Layer

**Figure 5:**
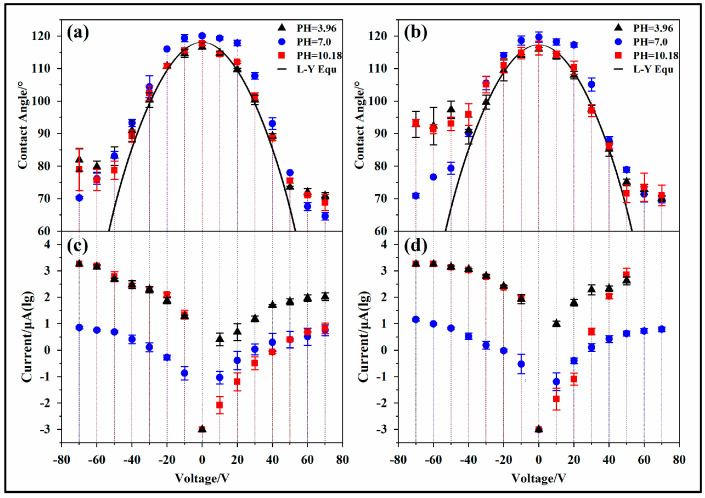
Effect of voltage on droplet contact angle and leakage current. Electrowetting models: (**a**,**c**) ITNAD and (**b**,**d**) ITN6D.

Regarding the experimental electrowetting models ITNAD and ITN6D, several interesting dielectric wetting phenomena could be observed. The test results are shown in Figure 5, where the black solid line represents the fitted value of the Lippmann–Young equation, and the initial contact angle was obtained via the average of the initial contact angles of three types of droplets: the black solid triangle indicates that the tested droplets are CA-SCB with a pH of 3.96; the blue solid circle indicates that the tested droplet is UP water with a pH of 7, and the red solid square indicates that the tested droplets are SC-SBB with a pH of 10.18.

1. Contact angle deviation

The measured value of the droplet contact angle with voltage variation deviates from the theoretical value. Additionally, as the voltage increases, the droplet contact angle gradually saturates. The deviation situation is shown in Table 3. In the ITNAD test model, the droplet contact angles are saturated at approximately 60 V and −50 V with saturated contact angles of 70° and 80°, respectively. In the ITN6D test model, the droplet contact angle is saturated at approximately 50 V and −40 V with saturated contact angles of 70° and 90°, respectively. It is worth noting that as the voltage increases, the measured contact angle deviates more from the theoretical value.

2. Contact angle asymmetry

The change in the droplet contact angle is influenced by the polarity of the voltage, and there is a clear asymmetric phenomenon along the axis V = 0. The experimental results of the ITNAD test model are shown in Figure 5a. The measured value of the droplet contact angle with voltage variation is inconsistent with the initial voltage deviation from the theoretical value. In the case of positive voltage polarity, the droplet contact angle gradually deviates from the theoretical value at about 50 V. In the case of negative voltage polarity, the droplet contact angle gradually deviates from the theoretical value at about 40 V. Meanwhile, the saturation contact angles of the positive and negative voltages are inconsistent, and the saturation contact angle with a positive voltage is generally 10 times smaller than that with a negative voltage. The experimental results of the ITN6D test model are shown in Figure 5b. In the case of positive voltage polarity, the droplet contact angle gradually deviates from the theoretical value at about 40 V. In the case of negative voltage polarity, the droplet contact angle gradually deviates from the theoretical value at about 30 V. Moreover, the saturated contact angle with a positive voltage is generally 20° smaller than that with a negative voltage.

3. Effect of pH

The contact angle of the droplets varies with voltage due to the influence of the droplet ion concentration and pH. Under a positive voltage, the three droplets with different pH values exhibit similar electrowetting behavior. However, under a negative voltage, the charged contact angle of 0.1 mol/L of ionic liquids (pH = 3.96 and pH = 10.18) is more likely to deviate from the theoretical value and reach a saturation state. The saturation contact angles are about 80° (ITNAD) and 90° (ITN6D), and the absolute saturation voltages are about 50 V (ITNAD) and 40 V (ITN6D). Under UP water (pH = 7) conditions, the saturated contact angle is below 70°, and the absolute saturation voltage is greater than 70 V. Interestingly, ionic liquids with pH = 3.96 and pH = 10.18 exhibit similar dielectric wetting behavior.

4. Effect of hydrophobic materials

The influence of the dielectric wetting models composed of different hydrophobic materials on the contact angle of the droplets is not consistent. Compared with the dielectric wetting models ITNAD and ITN6D, the contact angle of the ion droplets in the ITN6D wetting model is more likely to deviate from the theoretical value with voltage changes. The saturated contact angle of the droplets with negative voltage is 15°~20° larger than the former, and the saturation voltage is about 20 V lower than the former.

### 3.3. Leakage Current

The leakage current varies in the different electrowetting models. In the experimental wetting models ITKAD and ITK6D, the leakage current in the circuit is small, as shown in Figure 4c,d. When UP water is used as the tested droplet, the leakage current does not exceed 10 nanoamperes. When an ionic liquid is used as the tested droplet, the leakage current is relatively high, but the maximum does not exceed 35 nA. At the same time, the leakage current exhibits asymmetry, and the leakage current with a negative charge is one to three times higher than that with a positive charge.

In the experimental electrowetting models ITNAD and ITN6D, the leakage current in the circuit is relatively large, as shown in Figure 5c,d. The leakage current also exhibits voltage polarity asymmetry. When UP water is used as the tested droplet, the leakage current does not exceed 15 microamperes, and the leakage current with a negative charge is two to three times higher than that with a positive charge. When ionic liquids are used as the tested droplet, the maximum leakage current exceeds 1.8 mA. Among them, the leakage current of acidic droplets with a negative charge is 5~10 times higher than that with a positive charge, and the leakage current of alkaline droplets with a negative charge is three to four orders of magnitude (ITNAD) and one to five orders of magnitude (ITN6D) higher than that with a positive charge. It is worth noting that when the voltage was high, the droplets bounced, and, thus, the leakage current could not be measured. Therefore, some data points are missing in the figure.

In addition, in order to investigate the intrinsic properties of hydrophobic materials, we developed a test model consisting of an ITO–hydrophobic layer–gold layer and used the probe method to test the leakage current. The test results show that under a voltage of 10 V, the intrinsic properties of the two materials are similar, and the circuit current is in the range of 0.3~0.4 nA.

## 4. Discussion

### 4.1. Dynamics of Droplets in the Electric Field

**Figure 6:**
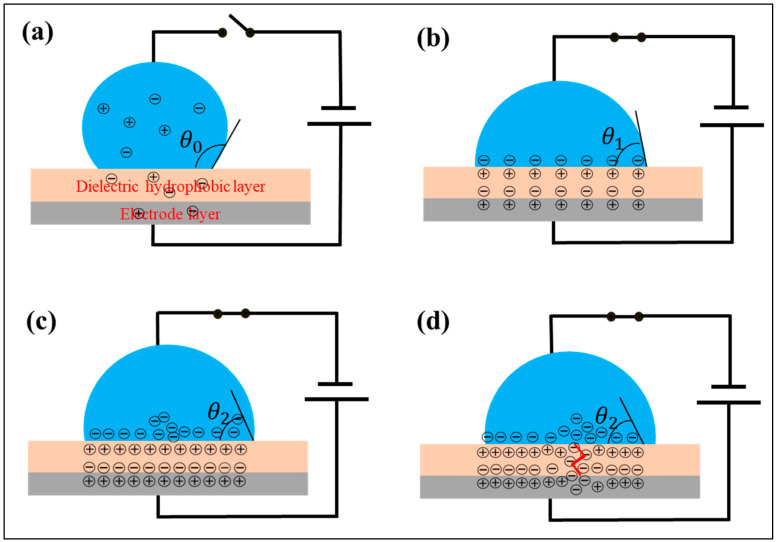
Four stages of droplet in dielectric wetting process: (**a**) initial stage; (**b**) normal polarization stage; (**c**) ion-trapping stage; (**d**) breakdown stage.

From a microscopic point of view, there are only a quite few free electrons in the droplet and the hydrophobic dielectric layer without electric charge, as shown in Figure 6a. Once charged, the dielectric wetting behavior of the droplet can be analyzed in three stages. The first stage is the normal polarization stage, as shown in Figure 6b. The same amount of dissimilar charges gather at the interface between the microdroplet and the dielectric layer as well as between the dielectric layer and the electrode layer to form a double electric layer. The second stage is the ion-trapping stage, as shown in Figure 6c. With the increase in electric field strength, more and more ions are adsorbed on the surface of the hydrophobic dielectric layer. The third stage is the breakdown stage, as shown in Figure 6d. With the further increase in electric field strength, the hydrophobic dielectric layer is broken down, and ions enter into the hydrophobic dielectric layer.

In addition, the droplet contact angle of the three stages varies with the voltage. With the increase in voltage, the three stages are gradually in transition states from the normal polarization stage to the ion trapping stage and then to the breakdown stage. The influence of the stage changing is also different, resulting in a complex law of contact angle change. And this problem can be analyzed from the point of view of an equivalent circuit.

### 4.2. Equivalent Circuit of the Thick Hydrophobic Layer

When the hydrophobic dielectric layer is thick (about 2.5 μm), the leakage current of acidic, alkaline and UP water is very small. The state of this part of the droplet is shown in Figure 6b, and the weight of the normal polarization stage is the highest. The equivalent circuit is analyzed as the following parts.

**Figure 7:**
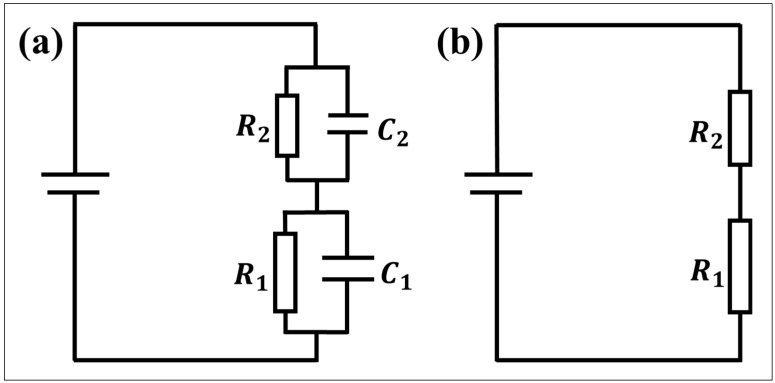
Equivalent circuit diagram of the electric wetting of the thick hydrophobic dielectric layer. (**a**) Ideal model. (**b**) Simplified model.

In the experimental electrowetting models ITKAD and ITK6D, the leakage current in the circuit, whether using ionic liquids or UP water, is measured to be less than 35 nA. This indicates that the dielectric performance of the dielectric layer is good and that it is difficult for electrons to move in the dielectric layer. Almost no electrochemical reactions occur in the droplets, while the conductivity of the tungsten wire and ITO electrode is strong, and the resistance can be ignored. Currently, the dielectric wetting model is equivalent to a series connection of the dielectric layer RC circuit and the droplet RC circuit, as shown in Figure 7a, where *R*_1_ and C_1_ represent the equivalent resistance and capacitance of the dielectric layer, and *R*_2_ and C_2_ represent the equivalent resistance and capacitance of the droplet with the charging method being DC charging. Therefore, the influence of capacitive impedance is not considered to be temporary. The model can be simplified, as shown in Figure 7b.

According to Table 2, the volume resistivity of AF1601 is *ρ*_1_ = 3.3 × 10^11^ Ω m, and the volume resistivity of 6%T6 is *ρ*_2_ = 2.9 × 10^11^ Ω m. As measured using a conductivity meter, the UP water resistance is *ρ*_3_ = 1.25 × 10^4^ Ω m. The resistivity of the CA-SCB solution is *ρ*_4_ = 0.97 Ω m. The resistivity of the SC-SBB solution is *ρ*_5_ = 1.92 Ω m. The calculation formulas for the spherical droplet volume and resistance are

(2) $$V=\frac{4\pi }{3}r^{3},$$

(3) $$R=\frac{\rho L}{S},$$

Among them, *V* represents the volume of the droplet, *r* represents the radius of the spherical droplet, *R* represents the resistance, *ρ* represents the resistivity, *S* represents the area, and *L* represents the length. In the process of dielectric wetting, it is assumed that the droplet maintains the standard hemispherical state, and the volume of the droplet tested in the experiment is 5 μL. According to Equations (2) and (3), the theoretical resistance of the AF1601 dielectric layer is *R*_1_ = 1.6 × 10^11^ Ω, and the theoretical resistance of the 6%T6 dielectric layer is *R*_2_ = 1.4 × 10^11^ Ω. The theoretical droplet resistance of UP water is *R*_3_ = 3.1 × 10^6^ Ω. The theoretical droplet resistance of the CA-SCB solution is *R*_4_ = 2.4 × 10^2^ Ω. The theoretical droplet resistance of the SC-SBB solution is *R*_5_ = 4.7 × 10^2^ Ω.

At this point, the resistance of the dielectric layer (*R*_1_ and *R*_2_) is much higher than the droplet resistance (*R*_3_, *R*_4_, and *R*_5_). Therefore, most of the voltage drop in the circuit occurs in the dielectric layer. By fitting the data on the leakage current and circuit voltage, the corresponding resistance values in the circuit under different conditions can be obtained. The results show that the measured value of the dielectric layer resistance is basically consistent with the theoretical calculated value, which is in the order of 10^11^. However, the droplet resistance is below the order of 10^6^, which is far less than the dielectric layer resistance. Therefore, the change in the droplet resistance has minimal impact on the voltage. In the absence of electrochemical reactions and damage to the dielectric layer, the actual electric field strength applied to both ends of the droplet is consistent with the theoretical electric field strength. Therefore, the change in the droplet contact angle with the voltage (−150~150 V) follows the Lippmann–Young equation, and no contact angle saturation or asymmetric electrowetting phenomena is found. It is also shown that the variation in droplet contact angle with voltage in the normal polarization stage is in accordance with the Lippmann–Young equation.

### 4.3. Equivalent Circuit of the Thin Hydrophobic Layer

When the hydrophobic dielectric layer is thin, the leakage current value of the droplet increases obviously and is strongly affected by pH. At this point, the weight of the ion trapping and breakdown stages increases, and their equivalent circuits are changed.

In the experimental electrowetting models ITNAD and ITN6D, according to Equation (3), the resistance of the dielectric layer is calculated to be 10^11^ Ω. However, through the theoretical fitting of the voltage and leakage current, it is found that the external circuit resistance value changes between 10^4^ and 10^11^, indicating that the dielectric layer resistance changes. This may result from the occurrence of electrochemical reactions and the porosity of thin layers changing the equivalent circuits.

On the one hand, electrochemical reactions occur in droplets (water is electrolyzed), in which hydrogen ions at the cathode gain electrons to generate hydrogen gas, and hydroxide ions at the anode lose electrons to generate oxygen and water. The total reaction formula is shown in Equation (4), and it is similar to the phenomenon observed by Mehdi Khodayari et al. [25]. At the same time, it is difficult for further oxidation reactions to occur in ITO under a positive voltage, because In and Sn are already in the highest valence states. Under a negative voltage, the indium ions in indium tin oxide are easily reduced to indium ions, which affects the hydrophobic dielectric layer. The total reaction formula is shown in Equation (5), and Senthilkumar [43] and Matveeva et al. [44] also found this phenomenon in acidic and alkaline liquids. Meanwhile, when a positive voltage is applied, the chemical properties of the tungsten wire used as the cathode are relatively stable. When a negative voltage is applied, the tungsten wire used as the anode is easily oxidized to form tungsten oxide. The total reaction formula is shown in Equation (6).

(4) $$2H_{2}O=2H_{2}+O_{2},$$

(5) $$2{\mathrm{In}}_{2}O_{3}=4\mathrm{In}+3O_{2},$$

(6) $$2W+3O_{2}=2{\mathrm{WO}}_{3},$$

On the other hand, the hydrophobic dielectric layer is not dense, but the layer presents a porous structure, as shown in the SEM images. As the voltage increases, the wettability of the droplets gradually increases and may penetrate into the porous structure of the hydrophobic layers. Therefore, in an equivalent circuit, the hydrophobic dielectric layer needs to be considered in terms of two parts: one part is the hydrophobic dielectric layer itself, which is equivalent to a fixed value resistor and a capacitor in parallel, and the other part is the interaction between the hydrophobic dielectric layer and the droplet, which is equivalent to a variable resistance. The shape of the droplet changes with the voltage and undergoes a hydrolysis reaction, which can be equivalent to a variable resistor and capacitor connected in parallel. The tungsten wire surface and ITO surface undergo oxidation–reduction reactions, respectively, which can also be equivalent to a variable resistor and capacitor in parallel.

**Figure 8:**
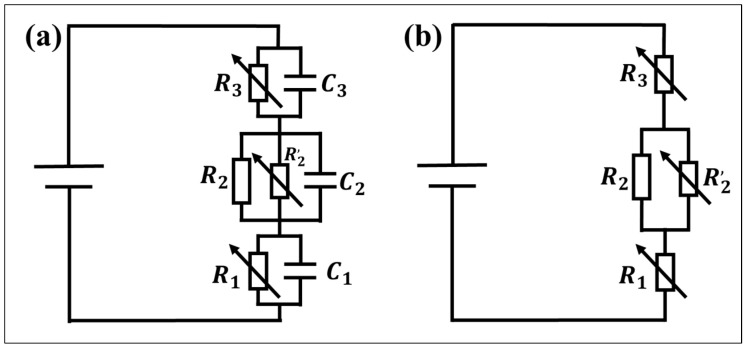
Equivalent circuit diagram of the electrowetting of the thin hydrophobic dielectric layer. (**a**) Ideal model. (**b**) Simplified model.

Based on the aforementioned research, we construct an equivalent circuit diagram of the electrowetting model composed of ITO–hydrophobic layer–droplet–tungsten wire, as shown in Figure 8a. In the diagram, *R*_1_ and C_1_ represent the equivalent resistance and equivalent capacitance of the ITO electrode and tungsten wire electrode. *R*_2_ and C_2_ represent the equivalent resistance and equivalent capacitance of the dielectric layer, and *R*_2_′ represents the pore resistance [45] (equivalent resistance of droplets penetrating the dielectric layer). *R*_3_ and C_3_ represent the equivalent resistance and equivalent capacitance of the droplets. The charging method used in this study is DC charging, so the influence of capacitive reactance is not currently considered. The model can be simplified, as shown in Figure 8b. By using this equivalent circuit, we attempt to investigate the electrowetting phenomenon on thin hydrophobic layers.

#### 4.3.1. Contact Angle Deviation

For UP water droplets, the free ion concentration is very low. As the voltage increases, the contact angle of the droplet deviates from the Lippmann–Young equation with the change in voltage, but it is symmetric with the polarity of voltage. At this point, the weight of the ion-trapping stage increases.

The deviation phenomenon where the measured droplet contact angle with voltage variation deviates from the theoretical value can be analyzed from pore resistance. As the voltage gradually increases, the wettability of the droplets increases, and they gradually seep into the dielectric layer. At this point, the equivalent resistance of the dielectric layer determined by *R*_2_ becomes *R*_2_*R*_2_′/(*R*_2_ + *R*_2_′), causing a sharp decrease in the total resistance of the dielectric layer from the original 10^11^ level to the 10^4^~10^7^ level. Pore resistance plays a dominant role and begins to be affected by the external circuit voltage. At the same time, as the voltage increases, the number of pores in the dielectric layer penetrated by the droplet increases, and the pore resistance decreases accordingly. Even if the voltage of the external circuit continues to increase, the voltage applied at both ends of the dielectric layer does not change, resulting in the actual electric field strength applied at both ends of the droplet not changing with voltage, thus exhibiting the contact angle saturation phenomenon.

#### 4.3.2. Contact Angle Asymmetry

For acidic and alkaline droplets, the free ion concentration is high. As the voltage increases, the contact angle of the droplet deviates from the Lippmann–Young equation, and the polarity of the voltage is obviously asymmetrical. At this point, the weight of the breakdown stage increases.

**Figure 9:**
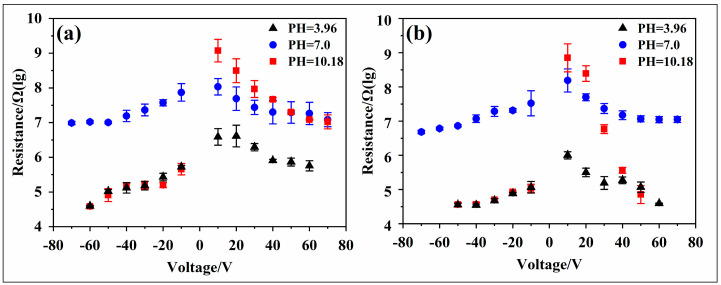
The relationship between the equivalent resistance of the circuit and voltage of the electrowetting model. Electric wetting models: (**a**) ITNAD; (**b**) ITN6D.

Based on the phenomenon of voltage polarity asymmetry, as the voltage increases, the electrochemical reaction in Equation (3) occurs in the droplet, resulting in the actual electric field strength added at both ends of the droplet being lower than the theoretical value. Through a fitting analysis of the leakage current and applied voltage, it is found that under the same external circuit voltage, the equivalent resistance of the circuit with a negative voltage is smaller than that with a positive voltage, as shown in Figure 9. Therefore, the leakage current in the circuit with a negative voltage is much greater than that with a positive voltage, and the electrochemical reaction is more intense. In the figure, the black solid triangle represents the resistance value of the circuit when the test droplet is CA-SCB, the blue solid circle represents the resistance value of the circuit when the test droplet is UP water, and the red solid square represents the resistance value of the circuit when the test droplet is SC-SBB.

In the case of the ITNAD electrowetting model, when the tested droplet is an acidic liquid, the equivalent resistance of the circuit with a negative voltage is about 10 times smaller than that of the circuit with a positive voltage. When the tested droplet is an alkaline liquid, the equivalent resistance of the ITNAD electrowetting model with a negative voltage is 100~1000 times smaller than that with a positive voltage. In the case of the ITN6D electrowetting model, the influence of voltage polarity on resistance is more significant. In particular, when the tested droplet is an alkaline liquid, the equivalent resistance of the ITN6D wetting model with a negative voltage is 10~10,000 times smaller than that with a positive voltage. This may be due to the oxidation–reduction reaction on the electrode surface, as shown in Equations (5) and (6), which occurs when a negative voltage is applied, resulting in an increase in the equivalent resistance (*R*_1_) of the electrode and a decrease in the partial voltage at both ends of the dielectric layer. Therefore, when a negative voltage is applied, the electric field strength at both ends of the ionic droplet is lower than that when a positive voltage is applied. The electrochemical reaction is the main reason for the polarity asymmetry of voltage electrowetting, and the leakage current is an important characteristic index.

#### 4.3.3. Effect of pH

The electrowetting phenomenon varies with the different pH of droplets. In Table 3, it can be observed that the conductivity of acidic liquids (σ_1_ = 0.52 S/m) is similar to that of alkaline liquids (σ_2_ = 1.03 S/m) but much higher than that of UP water (σ_3_ = 8 × 10^−5^ S/m). Therefore, the leakage current of acidic and alkaline liquids is much greater than that of UP water, and the electrochemical reaction occurring inside them is more intense. Through further analysis, it is found that the dielectric wetting behavior of acidic (pH = 3.96) and alkaline (pH = 10.18) droplets is similar at the same voltage, and their leakage current is almost the same. It is suggested that this effect is related to the total ion concentration of the droplet, not just the preferential adsorption of OH^−^ on the surface of the hydrophobic layer. Therefore, we believe that the migration of anions and cations in the hydrophobic dielectric layer is a more critical reason. In addition, the electrochemical reaction on the electrode surface also affects the migration of anions and cations.

#### 4.3.4. Effect of Hydrophobic Materials

It is worth noting that different hydrophobic dielectric materials also have different electrowetting phenomena. The surface morphology of the two materials was obtained using scanning electron microscopy (Figure 3), and the electrical resistivity of the two materials was measured using a conductivity meter (Table 2). This study found that the morphology, porosity, and electrical resistivity of the two materials were not significantly different, and the influence of these factors could be preliminarily ruled out. Meanwhile, by comparing the two hydrophobic materials 6%T6 and AF1601, further research found that the contact angle deviation phenomenon and asymmetric electrowetting phenomenon of 6%T6 were more obvious, as shown in Figure 5a,b. In addition, it can be seen from Figure 5c,d that under the same conditions, the leakage current in AF1601 is greater than that in 6%T6. Therefore, we speculate that the mobility of anions and anions in the two materials is different. In AF1601, the mobility of anions and anions is similar. In 6%T6, the mobility of cations is higher than that of anions, and this difference results in the different asymmetric curve of the two materials.

## 5. Conclusions

According to the principle of dielectric wetting, the dielectric wetting phenomenon varies among different hydrophobic dielectric layers and droplets. When the thickness of the hydrophobic dielectric layer (AF1601 and 6%T6) is about 2.5 μm, the dielectric performance is good, and the maximum leakage current does not exceed 35 nA. At this point, the contact angle of acidic, UP water and alkaline droplets all follow the Lippmann–Young equation with voltage (−150~150 V), and no asymmetric electrowetting phenomenon occurs. When the thickness of the hydrophobic dielectric layer (AF1601 and 6%T6) is about 340 nm, the dielectric performance is poor, and the leakage current is relatively large. The contact angle of acidic, UP water and alkaline droplets all follow the Lippmann–Young equation with voltage (−40~40 V). In the voltage range of −70~−40 V and 40–70 V, when the droplet is UP water, and the droplet contact angle deviates from the theoretical value with the change in voltage, it is symmetric with the polarity of voltage. When the droplet is acidic or alkaline, the contact angle of the droplet deviates from the theoretical value with voltage variation, but it is asymmetrical with the polarity of voltage.

In the process of dielectric wetting, the dielectric wetting behavior of the droplet can be divided into three stages. The first stage is normal polarization state. At this point, the leakage current of the droplet is very small, and the change in the droplet contact angle with voltage conforms to the Lippmann–Young equation. The second stage is the ion-trapping state. At this stage, the leakage current increases, and the droplet contact angle deviates from the Lippmann–Young equation with the change in voltage but is symmetric with the polarity of voltage. The third stage is the breakdown state. At this point, the leakage current of the droplet further increases, and the droplet deviates from the Lippmann–Young equation with voltage variation, and the asymmetric electrical wetting phenomenon of voltage polarity is generated. Moreover, it can be seen from the leakage current that the main source of asymmetry is the different mobility of anions and cations to the hydrophobic dielectric layer, which is closely related to the intrinsic properties of the hydrophobic dielectric materials.

In addition, the dielectric wetting behavior of acidic droplets and alkaline droplets is similar with significant differences in the dielectric wetting behavior between thick and thin hydrophobic dielectric layers. The reason for this phenomenon is not only the adsorption of hydroxide ions on the surface of hydrophobic layers as reported in the literature, but also the properties of hydrophobic materials and the types of ions in droplets may be a more important influencing factors, which are also the subject of continuing analytical investigation.

## Figures and Tables

**Table 1:** Reagent formula.

Reagent	CA-SCB		SC-SBB	
	Citric Acid	Sodium Citrate	Sodium Carbonate	Sodium Bicarbonate
Ratio	3.8	16.2	6	4
Conc (mol/L)	0.1	0.1	0.1	0.1
Target pH value	4.0		10.0	

**Table 2:** Four types of EWOD models.

EWOD Models	Hydrophobic Layers			Droplets		
	Type	δ (nm)	*ρ* (Ω m)	Type	pH	σ (S/m)
ITKAD	AF1601	2489 ± 35	3.3 × 10^11^ ± 10^10^	CA-SCB	3.96 ± 0.12	1.03 ± 0.05
				UP water	7.00 ± 0.32	8 × 10^−5^ ± 10^−6^
				SC-SBB	10.18 ± 0.14	0.52 ± 0.03
ITNAD	AF1601	344 ± 2	1.9 × 10^12^ ± 5 × 10^10^	CA-SCB	3.96 ± 0.12	1.03 ± 0.05
				UP water	7.00 ± 0.32	8 × 10^−5^ ± 10^−6^
				SC-SBB	10.18 ± 0.14	0.52 ± 0.03
ITK6D	6%T6	2501 ± 176	2.9 × 10^11^ ± 9 × 10^9^	CA-SCB	3.96 ± 0.12	1.03 ± 0.05
				UP water	7.00 ± 0.32	8 × 10^−5^ ± 10^−6^
				SC-SBB	10.18 ± 0.14	0.52 ± 0.03
ITN6D	6%T6	343 ± 12	2.0 × 10^12^ ± 0	CA-SCB	3.96 ± 0.12	1.03 ± 0.05
				UP water	7.00 ± 0.32	8 × 10^−5^ ± 10^−6^
				SC-SBB	10.18 ± 0.14	0.52 ± 0.03

**Table 3:** Deviation degree of droplet contact angle under different voltages.

Reagent Type	Voltage (V)	Measured Value–Theoretical Value (°)				
		−70~−60 V	−50~−40 V	−30~30 V	40~50 V	60~70 V
CA-SCB	ITNAD	>42	~10	~3	~6	>34
	ITN6D	>54	~19	~2	~5	>35
UP water	ITNAD	>34	~10	~3	~10	>26
	ITN6D	>33	~6	~3	~4	>28
SC-SBB	ITNAD	>37	~8	~3	~5	>32
	ITN6D	>53	~19	~2	~3	>35

## Data Availability

Data are contained within this article.
